# Resistance to Resilience: Understanding Post-surgical Hormone Therapy in Breast Cancer Care

**DOI:** 10.7759/cureus.47869

**Published:** 2023-10-28

**Authors:** Tanishq Kumar, Rajoshee R Dutta, Swedaj Thakre, Arihant Singh, Vivek R Velagala, Raju K Shinde

**Affiliations:** 1 Medicine, Jawaharlal Nehru Medical College, Datta Meghe Institute of Higher Education and Research, Wardha, IND; 2 General Surgery, Jawaharlal Nehru Medical College, Datta Meghe Institute of Higher Education and Research, Wardha, IND

**Keywords:** aromatase inhibitors, tamoxifen, adherence, breast cancer, hormone therapy

## Abstract

Breast cancer is one of the most common types of cancer affecting women worldwide. Over the years, breast cancer has become a major public health concern, and its incidence is rising globally. The treatment of breast cancer does not stop with surgical intervention, but adjuvant therapies are administered to improve patient outcomes post-surgery based on the type of breast cancer diagnosed. This review focuses on the value of hormone therapy (HT) in improving the prognosis of breast cancer patients and why adhering to adjuvant treatment post-surgery is difficult for patients. HT aims to reduce the chances of breast cancer recurrence after surgical treatment. Even though HT is life-saving, patients tend to not adhere to the therapy due to various factors such as side effects, age-related issues, and socioeconomic status. Most patients stop adhering to the therapy as the duration can be as long as 5-10 years, and the quality of life is greatly impacted due to the side effects of the treatment. This review examines the possible factors leading to non-adherence to HT and tries to propose possible interventions that might improve patient compliance with the treatment. This article not only focuses on the impact of side effects of HT on patients' quality of life but also tries to understand the problems faced by breast cancer patients in adhering to HT.

## Introduction and background

Breast cancer is one of the most prevalent forms of cancer affecting women all over the world. Its incidence varies globally and is found to be lower (below 50 per 100,000) in the majority of developing countries compared to developed countries where the incidence is well above 50 per 100,000 people [1]. Even though the incidence is high in developed nations, advancements in screening, early detection, and treatment have reduced mortality rates in these patients. Based on a report in 2019, the death rate due to breast cancer in North America was 12.6 per 100,000 while 13.36 per 100,000 in Europe [2]. In contrast, the mortality rate in Central and Eastern Europe and Africa was approximately 15.5 per 100,000 and 18.4 per 100,000 respectively [1].

**Figure 1:**
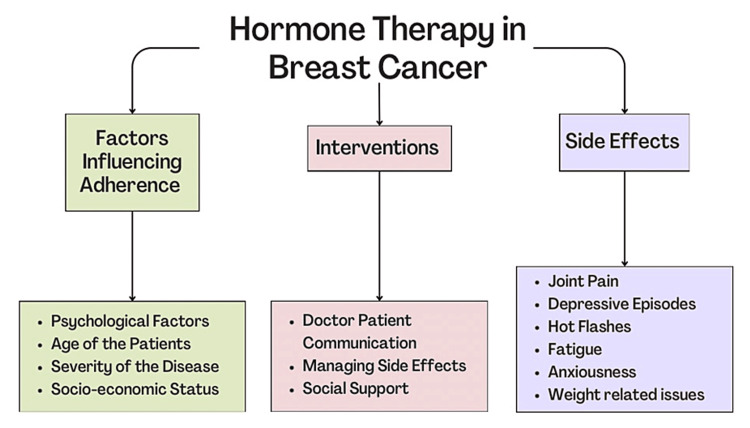
Summary of factors influencing adherence, side effects, and interventions related to hormone therapy for breast cancer Image credits: Tanishq Kumar

The mortality rates have been low compared to the incidence rates due to continuous breakthroughs in treating and managing breast cancer. One such advancement has been the development of long-term hormone therapy (HT) for patients who are hormone receptor (HR)-positive cases, constituting a major proportion of breast cancer-diagnosed cases [3]. The most common drugs used in endocrine therapy (ET) are tamoxifen or an aromatase inhibitor (AI). Adjuvant treatment with HT post-surgery has proved to reduce the recurrence and mortality rates in breast cancer patients [4,5]. This article examines the factors affecting adherence to HT, its side effects, and the interventions that can be made to improve adherence to HT in breast cancer patients. Figure 1 provides a summary of factors affecting adherence to treatment, side effects, and interventions to improve adherence.

## Review

Methodology

Eligibility Criteria

The eligibility criteria included articles that discussed factors affecting adherence to HT. Articles focusing on the side effects of HT, and interventions that can improve adherence to HT in breast cancer management were also included. Articles that focused only on the treatment of breast cancer were excluded. There were no other exclusion criteria.

Literature Search Strategy

All authors were involved in the literature search. The PubMed electronic database was used for the literature search. We searched for relevant articles published from the year 1999 to the year 2022. The following key terms were used in the search: "Hormone Therapy," "Breast Cancer," “Adherence,” “Tamoxifen,” and “Aromatase inhibitors.”

Data Extraction

**Figure 2:**
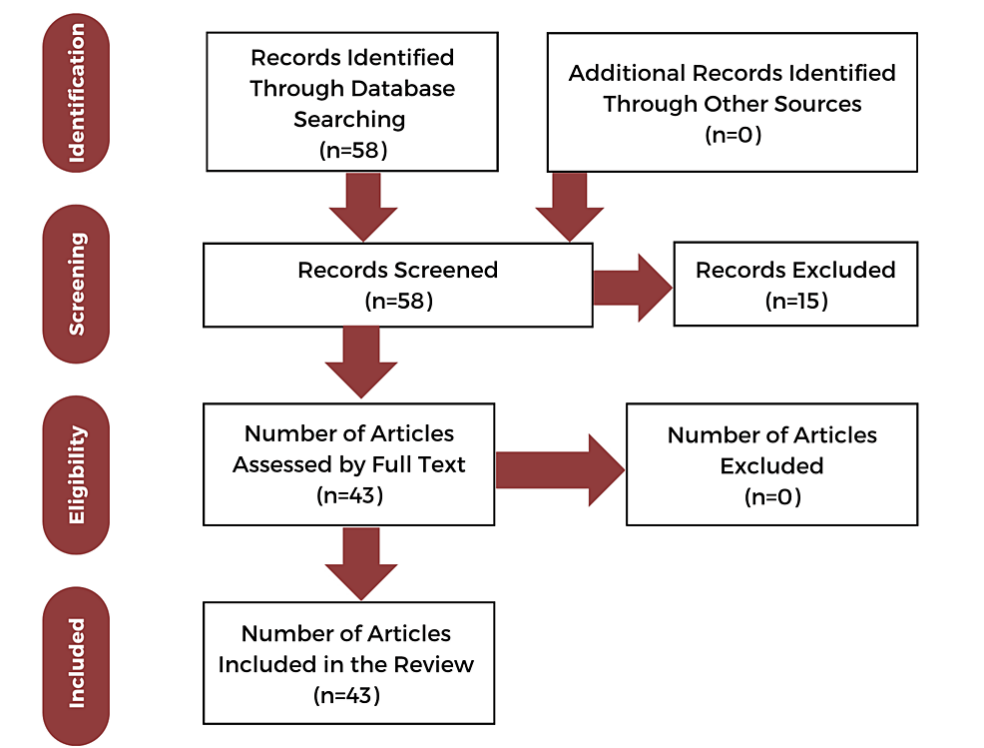
PRISMA flow diagram depicting the screening and selection of articles PRISMA: Preferred Reporting Items for Systematic Reviews and Meta-Analyses

The abstracts of the articles generated by the literature search were reviewed by all authors independently. Those that met the selection criteria were studied and assessed for their full texts. The search process is illustrated in the flow chart in Figure 2.

Hormone therapy

HT is used for treating hormone-receptor-positive breast cancer cases, which account for nearly 85% of all types of breast cancer [6]. HT aims to stop any interaction between estrogen and estrogen-based biochemical reactions that might trigger cancer cells [7]. HT is based on blocking signaling between estrogen and tumor cells. Disruption of estrogen receptors occurs either by directly binding to the receptor, which can be observed in estrogen receptor agonists/antagonists (ERAAs) drugs like tamoxifen, or by diminishing estrogen synthesis by AIs like letrozole, anastrozole, and exemestane. One of the most common drugs under use for both pre and postmenopausal patients is tamoxifen [7]. Nearly all female patients with HR-positive breast cancer are advised to undergo HT. If the regimen is strictly followed, HT can potentially lower breast cancer recurrence by 40% and the death rate by almost a third [8]. When HR-positive breast cancer is diagnosed, usually HT is initiated except in cases when adjuvant chemotherapy is also to be given. In such scenarios, HT starts after chemotherapy is over. Based on recurrence risk and the type of HT regimen, the ideal treatment duration could be around 5-10 years. Even though the benefits of HT are many, non-adherence to the therapy is reported to be very high. Nearly 50% of the women administered with HT do not take 100% of their recommended dose [9,10]. Almost 50% of the patients stop adhering to the regimen in the fifth year of starting their therapy [11,12].

Adherence and non-adherence

Adherence, in medical terms, is defined as the degree of compliance exhibited by a patient in following an agreed disease management prescribed by a healthcare professional [13]. Not complying with the approved treatment plan by making lapses either by missing or taking extra doses of the prescribed medications is termed as non-adherence. Non-adherence can be classified based on behavior into types: unintentional and intentional non-adherence. Unintentional non-adherence could be due to a confusing medication plan, poor understanding of the doses, or physical or mental disability. On the other hand, the intentional decision to avoid the prescribed medical management comes under intentional non-adherence [14]. According to a World Health Organisation report, if a patient's adherence level ranges from 80% to 120% with regard to the recommended regimen for a considerable duration, then that patient can be put under the adherent category [13].

Side effects of hormone therapy

Side effects of HT are one of the main reasons for non-adherence to it. These side effects not only reduce the quality of life on a daily basis but also have some psychological as well as physical implications. Some of the most common side effects are joint pain/osteoporosis, depressive episodes, hot flashes, difficulty sleeping, fatigue, anxiousness, and weight-related issues [15-21]. Poor knowledge about these side effects and lack of constant support to the patients lead to non-adherence to HT, defeating the rationale behind initiating HT [12].

Hobson's choice or on the horns of a dilemma

A study revealed that most HR-positive breast cancer patients give consent to HT because they think they have no other treatment options. The risk of recurrence and the mandatory nature of the treatment regimen for HR-positive breast cancer leaves the patients with no choice. This scenario is called Hobson's choice. It means when there is no other feasible option with similar outcomes. Sometimes, this proves to be beneficial for some patients as a large number of patients follow the entire regimen till its completion, whereas knowledge about the side effects after initiating the HT or having their own experience with the side effects gives rise to a horned dilemma. It refers to having two similar negative options, making the decision to opt for any of them difficult. One possibility is to start the HT and endure the side effects till the prescribed duration. Another option is to not start the therapy and take their chances with regard to the recurrence of tumor cells [1].

A study based on interviews revealed that a segment of patients adhere to the prescribed therapy believing they are left with no option (Hobson's choice). At the same time, patients who are skeptical about HT experience a dilemma (horned dilemma) after enduring the side effects of the therapy. Eventually, due to the side effects, what started as Hobson's choice now puts the patient in a dilemma of reconsidering their decision to adhere to HT. The change in the outlook towards the therapy arises due to the side effects now overshadowing the benefits of the therapy, leading them to non-adherence to HT [22].

Factors influencing adherence to hormone therapy

Psychological Factors

Several studies have shown that the issue of non-adherence to the prescribed regimen is not simple. It does not solely originate from disease management, economic status, anthropometric measurements, or the different healthcare modalities. Now, it is understood that non-adherence is complex and difficult to quantify [23]. Psychological characteristics of the individual patient also contribute to the compliance or non-compliance towards the regimen. Some of the psychological perceptions related to HT include prescribed treatment plans, quality of life, effect on mental health, risk of recurrence, and patient-doctor relationship. Patients tend to fall into the dilemma of whether HT should be continued to avoid recurrence or discontinued, thereby risking relapse of cancer. These factors tend to influence adherence and non-adherence of patients toward HT in breast cancer [24].

Based on the findings of several studies, patients' personal viewpoints regarding the regimen prescribed by healthcare professionals are conflicted. The weighing of benefits against the side effects constantly causes confusion in adhering to the regimen [17]. A study showed that patients did not believe in the drugs' efficacy, leading to non-adherence [25]. Another study found that around 17% of patients did not follow the regimen after two years since they were not optimistic about the treatment, and side effects outweighed the benefits. In this group, patients gave more value to the quality of life. They were ready to face the risk of recurrence and probably shortened life span, too, rather than experiencing severe side effects of HT. [26]. On the other hand, patients who were more knowledgeable about the benefits followed the treatment plan involving tamoxifen and endured the side effects. This group was found to be more adherent to the hormone therapy. Managing the side effects by prescribing more drugs might play a role in contributing to non-adherence. A study found that emotions like optimism, self-belief, faith in HT, and trust in the healthcare personnel proved to be of key importance and contributed significantly to such adherent patients reporting fewer side effects of psychological illness than non-adherent patients [17].

Age of the Patients

The age of the patients also plays a role in adherence and non-adherence to the HT. Studies have revealed that patients who were very young or very old were non-adherent to HT [27]. This could be due to different age-related complications or expectations. In young patients, fear of losing fertility and daily hardships due to side effects of HT could contribute to non-adherence [28]. Poor quality of life in young patients would contribute to psychological illness and loss of faith in the treatment, leading to non-adherence to HT. [29]. On the other hand, old age comes with its own problems, such as chronic diseases, lack of proper knowledge regarding HT, poor social support, and lower motivation [30,31]. These studies suggest that patients at extreme ends of the age spectrum are at higher risk of being non-adherent to HT. Hence, healthcare workers should focus more on such patients and try to solve their misconceptions, doubts, or any modifiable concerns that might lead to positive outcomes for the patient.

Severity of the Disease

Patients suffering from terminal stages tend to adhere more to HT [32]. Sometimes, high-dose hormone therapy influences the patients' thinking and makes them realize their condition might be serious; therefore, such patients adhere more to HT. This might explain the higher degree of compliance seen in patients undergoing chemotherapy compared to patients who do not go through chemotherapy [33]. Sometimes, a high dose of HT might influence the patients towards non-adherence due to the side effects affecting the quality of life. Therefore, it is subjective to comment regarding patients' perception of the nature of HT and the stage of the disease [34].

Social and Economic Status

One of the most important aspects of any treatment is the patient's socioeconomic status, as the burden of chronic disease management might pose the greatest hurdle for the patient's management. Socioeconomic factors like financial situation, literacy, and social well-being of the patient actively influence the compliance and outcome of the treatment. Based on a study, it was found that lower or no payment for HT increased the compliance of the patients [35]. Another study found that even after receiving free medications for treatment, patients still did not follow the prescribed regimen. This suggests that socioeconomic status is not the sole reason patients do not fully comply with HT [36].

Interventions to improve adherence

Communication Between Doctors and Patients

Doctor-patient relationship in chronic disease management plays an important role in the outcome of the disease. A good professional relationship between the healthcare provider and the patient ensures great disease management. Based on a study, it was found that patients tend to remain adherent to HT if the doctor-patient relationship is good. A good relationship can be achieved when a doctor provides easy access to medical support, periodic assessment of the effectiveness of HT, and constant psychological support [37]. A study found that sometimes doctors unintentionally fail to elaborate on the chronic HT treatment plan and do not emphasize the side effects of the treatment and how it will influence the quality of life. Therefore, it is the duty of the doctor to make sure that patients have a complete understanding of their disease and HT [38]. It has been proved that patients with poor knowledge regarding HT, the potential side effects, and the impact on the quality of life tend to become non-adherent to HT [39,40]. On the other hand, patients who were well-oriented about the treatment plan and its side effects showed higher degrees of adherence to HT [41]. Therefore, good doctor-patient communication is often associated with effective adherence to HT [42].

Managing Side Effects

HT comes with its benefits and side effects. For some patients, side effects outweigh the benefits of the HT, and they become non-adherent to the treatment. Therefore, healthcare personnel should focus on managing the side effects, which will increase the rate of patients complying with HT in breast cancer management [12]. 

Social Support

A recent study focusing on understanding the impact of social support in breast cancer patients found that social support improves the degree of adherence to HT. Social support in the form of information circulated by healthcare providers increased their knowledge regarding the treatment plan, side effects, benefits, and positive outcomes of HT on complete adherence. Empathy, positivity, and sympathy from members of the family, friends, and cancer support groups help to motivate the patient to complete HT [43].

Future Studies and Scope of Research

Future studies can focus on the behavioral psychology of patients in adhering to HT. Various reasons for adherence or non-adherence based on behavioral patterns can be understood. Studies based on improving the quality of life can lead to long-term HT adherence [27].

## Conclusions

Breast cancer is a highly prevalent global health issue affecting people in all countries with varying incidence rates. Even though incidence rates are higher in developed nations compared to developing economies, these countries have lower mortality rates due to advanced screening tools, early detection, and disease management. One breakthrough in improving the outcomes for breast cancer survivors has been the introduction of HT, which significantly reduces recurrence and death rates if the patients adhere to the treatment regimen. However, several patients tend to become non-adherent to HT. This non-adherence is a culmination of several factors, such as side effects of the therapy, patient beliefs about the regimen, psychological implications of the treatment, disease severity, and socioeconomic status of the patients. Patients end up having a Hobson's choice due to no alternative treatment options, forcing them to start HT despite its side effects. After initiating the treatment, the patients face a horned dilemma due to the side effects influencing their daily lives. This confuses patients as they compare the positive against the negative aspects of the treatment and ponder whether they should continue the treatment or not.

Interventions such as effective doctor-patient communication, which help patients understand their treatment plan better as well as HT's benefits and side effects, will certainly increase adherence to HT. Healthcare providers should try to manage the side effects as much as possible to improve patient regimen compliance. Social support constituting family, friends, and cancer survivor groups increases patient's adherence to HT and enhances their chance of avoiding cancer recurrence. In this battle against the rising prevalence of breast cancer, issues related to HT should be addressed so that patients can reap the full benefits of this treatment. Research studies focusing on minimizing the side effects of HT or newer modalities of managing these side effects should be undertaken. Creating a protective bubble around the patients where doctors help them understand the disease better, social support giving them confidence, and continuous research in tackling breast cancer will surely increase our chances of winning the battle against breast cancer globally.
